# Biological Variability and Impact of Oral Contraceptives on Vitamins B_6_, B_12_ and Folate Status in Women of Reproductive Age

**DOI:** 10.3390/nu5093634

**Published:** 2013-09-16

**Authors:** Jennifer O. McArthur, HoMan Tang, Peter Petocz, Samir Samman

**Affiliations:** 1 Discipline of Nutrition and Metabolism, School of Molecular Bioscience, The University of Sydney, Sydney, NSW 2006, Australia; E-Mails: jennifer.mcarthur@sydney.edu.au (J.O.M.); htan5514@uni.sydney.edu.au (H.T.); 2 Department of Statistics, Macquarie University, Ryde, NSW 2112, Australia; E-Mail: peter.petocz@mq.edu.au

**Keywords:** vitamin B_6_, vitamin B_12_, folate, variability, oral contraceptives, women

## Abstract

Vitamins B_6_, B_12_ and folate play crucial metabolic roles especially during the reproductive years for women. There is limited reporting of within-subject variability of these vitamins. This study aimed to determine the within and between subject variability in serum vitamins B_6_, B_12_, folate and erythrocyte folate concentrations in young women; identify factors that contribute to variability; and determine dietary intakes and sources of these vitamins. Data were obtained from the control group of a trial aimed at investigating the effect of iron on the nutritional status of young women (age 25.2 ± 4.2 year; BMI 21.9 ± 2.2 kg/m^2^). The coefficients of variability within-subject (CV_I_) and between-subject (CV_G_) for serum vitamins B_6_, B_12_ and folate, and erythrocyte folate were calculated. Food frequency questionnaires provided dietary data. CV_I_ and CV_G_ were in the range 16.1%–25.7% and 31.7%–62.2%, respectively. Oral contraceptive pill (OCP) use was associated (*P* = 0.042) with lower serum vitamin B_12_ concentrations. Initial values were 172 ± 16 pmol/L and 318 ± 51 pmol/L for OCP and non-OCP users, respectively; with differences maintained at four time points over 12 weeks. BMI, age, physical activity, alcohol intake and haematological variables did not affect serum or erythrocyte vitamin concentrations. Vitamin B_12_ intakes were derived from traditional and unexpected sources including commercial energy drinks. Young women using OCP had significantly lower serum vitamin B_12_ concentrations. This should be considered in clinical decision making and requires further investigation.

## 1. Introduction

Vitamins B_6_, B_12_ and folate play crucial inter-related roles in DNA synthesis throughout the lifecycle especially during childhood, adolescence and the reproductive years for women [1,2,3]. Studies have shown an inverse correlation between the intake of folic acid during pregnancy and infants born with neural tube defects [4,5]; a higher incidence of neurological disorders when vitamin B_12_ deficiency persists [6]; and anaemia, depression and confusion present with vitamin B_6_ deficiency [3]. The concentrations of the vitamins in the circulation reflect an individual’s storage of these vitamins and their dietary intakes. For vitamin B_12_, intakes are determined by the predominant animal sources available to a population and the preferences of individuals [7,8]. While investigating the effects of meat or iron supplementation on biomarkers of nutritional status, we identified a failure of this population to meet dietary recommendations for vitamins B_6_, B_12_ and folate [9]. Accordingly concern over meeting intake benchmarks is justified particularly for vitamin B_12_ as vegetarianism is increasing in popularity among teenagers [10] with the prevalence of vegetarian tendencies amongst female adolescents being as high as 37%. As vitamin B_6_ is not limited to animal foods, there is some lessening of the impact of this trend. The vitamin B_6_ non-animal sources include processed cereals, fruits and vegetables [11]. Folate fortification of baked goods is supplementing the intakes of high folate foods such as green leafy vegetables. Whether the predominant dietary sources of vitamins B_6_, B_12_ and folate for young women are reflective of the foods analysed as having a high content of these vitamins is uncertain.

The analysis of control group data from a recently completed trial [9] will enable us to explore the within- and between-subject variability of vitamins B_6_, B_12_ and folate concentrations in women of reproductive age. The availability of data on diet and aspects of lifestyle will help to identify potential determinants of variability in vitamin concentrations.

## 2. Experimental Section

### 2.1. Participants

Healthy active women (18–35 years) taking no medications, other than OCP, or vitamin and mineral supplements, were enrolled in a randomized controlled trial that was investigating the impact of diet or supplements on the iron status of women of reproductive age [9]. All procedures involving human subjects were approved by The University of Sydney Human Ethics Review Committee. Written, informed consent was obtained from all volunteers prior to their participation in the study.

### 2.2. Blood Collection and Analysis

Blood samples were collected from all volunteers initially (week 0) then at four weekly intervals (weeks 4, 8 and 12). All samples were taken between 0730 and 0930 h from an antecubital vein. Veni-puncturists were from a single collection centre with pre-set protocols for collection and sample handling. The potential pre-analytical variation was minimised, *i.e.*, the women were in the fasted state (10 to 12 h), and reported not to have engaged in vigorous activity in the preceding 12 h, and did not consume alcohol for 24 h prior to the blood collection. Subjects were in the supine position during the blood collection. On each occasion, blood samples were collected into vacutainer tubes (Becton Dickinson, Franklin Lakes, NJ, USA): untreated tubes for the analysis of serum folate and vitamin B_12_ concentrations, and EDTA-coated tubes for the analysis of erythrocyte folate and serum vitamin B_6_ concentrations. Samples that were destined for the analysis of vitamin B_6_ were collected in tubes that were shielded from light. All blood samples were kept on ice for up to 2 h, and centrifuged at 1500 g for 10 min at 5 °C.

The concentrations of vitamin B_12_, serum- and erythrocyte-folate were determined using an automated system (UniCel DxI Immunoassay System, Beckman Coulter Inc., CA, USA). Plasma vitamin B_6_ (pyridoxal-5-phosphate) concentrations were determined using an HPLC method (Chromsystems Instruments and Chemicals GmbH, Munich, Germany). The inter-assay CV for vitamins B_6_, B_12_ and folate were 6%, 11.2% and 12.8%, respectively. The reference intervals were: serum vitamin B_6_, 35–110 nmol/L; serum vitamin B_12_, 150–750 pmol/L; serum folate, 8–25 nmol/L; and erythrocyte folate, >550 nmol/L [12].

### 2.3. Dietary and Exercise Data

A validated FFQ [13] was used to collect dietary data on 2 occasions. The first occasion (week 0) was at study commencement, reporting on dietary intake for the previous 12 weeks, and the second was at study conclusion (week 12). Intakes of vitamins B_6_, B_12_ and folate were examined further and the highest five contributing foods for each vitamin were determined. To achieve this for individual participants, the contributing foods for each of the three vitamins were identified and ranked. The participants reported their activity levels using the validated International Physical Activity Questionnaire Short Form [14]. The frequency and duration of activity enabled the calculation of the metabolic equivalent of task (MET).

### 2.4. Statistical Analyses

The variability in vitamin concentrations is influenced by the intrinsic biological variation (within-subject variance) expressed as the coefficient of variation (CV_I_); and the variance of the means among subjects (between-subject variance) expressed as the coefficient of variation (CV_G_). Biological variations were reported as CV_I_, CV_G_, index of individuality (CV_I_/CV_G_) and reference change value (RCV). The RCV is a measure of difference used to monitor serial data and is known as the critical difference [15]. Coefficients of variation (CV) were calculated using Minitab statistical software [16]. All other statistical calculations were carried out using SPSS [17]. Repeated-measures analyses were carried out for the longitudinal vitamin data, including possible explanatory variables as factors (e.g., OCP use) or covariates (e.g., BMI). Regression analysis was used to identify possible relationships between serum vitamin B_6_, vitamin B_12_ and folate and erythrocyte folate concentrations with the equivalent dietary vitamin intake, alcohol intake, BMI, age, MET, OCP use and haemoglobin concentrations. A probability value of *P* < 0.05 was set for statistical significance.

## 3. Results

Complete data sets were available for 22 participants, age 25.2 ± 4.2 (mean ± SD) years and BMI 21.9 ± 2.2 kg/m^2^. Alcohol consumption ranged from 0 to 75.7 g/day with median intakes of 5.5 and 3.0 g/day at weeks 0 and 12, respectively. Physical activity ranged from 4.0 to 240 MET h/week with median activity of 33.6 and 33.3 MET h/week at weeks 0 and 12, respectively. Mean corpuscular volume (MCV) and haemoglobin (Hb) levels were collected at each time period. There were no significant differences between OCP users or non-OCP users for MCV (89.1 ± 2.7 fL and 90.4 ± 3.5 fL) or Hb (127 ± 6 and 132 ± 9 mmHg).

### 3.1. Blood Analyses

**Figure 1:**
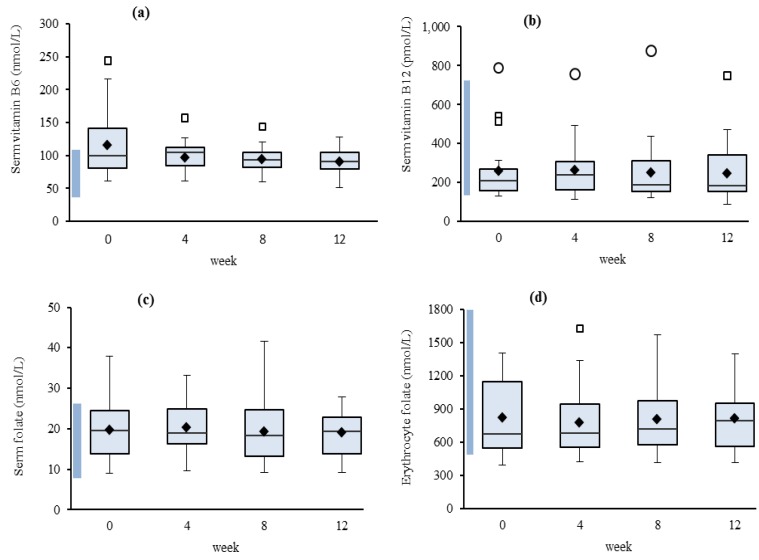
Mean **s**erum vitamins B_6_ (**a**), vitamin B_12_ (**b**), folate (**c**), and erythrocyte folate (**d**) concentrations (*n* = 22).^.^Reference intervals (shaded) [12], mean (♦) and outliers (□, ○)

A number of participants had vitamin B_6_ concentrations higher than the reference interval and the mean vitamin B_6_ concentration at week 0 was significantly higher than week 12 (*P* = 0.005) (Figure 1a). No other differences between mean values for any of the vitamins were noted.

The interquartile range for vitamin B_6_ demonstrated a spread at week 0 which decreased at subsequent sampling points (Figure 1a). The median vitamin B_12_ concentrations were in the lower 8%–22% of the reference interval, increasing in week 4. Frequency distributions for vitamin B_12_ were positively skewed and had two of the collections multi-modal (weeks 8 and 12) (Figure 1b). Serum- and erythrocyte-folate concentrations demonstrated similar characteristics. Serum folate demonstrated the greatest symmetry despite being multi-modal for two time-points (Figure 1c). Of the four analytes, erythrocyte folate had the largest interquartile range at week 0 (Figure 1d).

#### 3.1.1. Variability

The between-subject biological variations for the analysed vitamins were greater than the within-subject variations (Table 1) and the indices of individuality (CV_I_/CV_G_) were in the range of 0.26 (vitamin B_12_) to 0.81 (vitamin B_6_). The RCV at 49.8%–72.8% are influenced by the large within-subject variations of these vitamins. Maximum variances for each of the vitamins were 174% (vitamin B_6_; participant viii), 114% (vitamin B_12_; participant xvi), 153% (serum folate; participant x); and 147% (erythrocyte folate; participant i).

**Table 1:** CV_I_, CV_G_ and RCV for the serum vitamins B_6_, B_12_ and folate; and erythrocyte folate (*n* = 22).

	CV_G_ %	CV_I_ %	CV_I_/CV_G_	RCV (95%)
Serum				
Vitamin B_6_ (nmol/L)	31.7	25.7	0.81	72.8
Vitamin B_12_ (pmol/L)	62.2	16.1	0.26	49.8
Folate (nmol/L)	36.8	20.4	0.55	60.7
Erythrocyte				
Folate (nmol/L)	39.1	16.1	0.41	49.8

#### 3.1.2. Relationships

**Figure 2:**
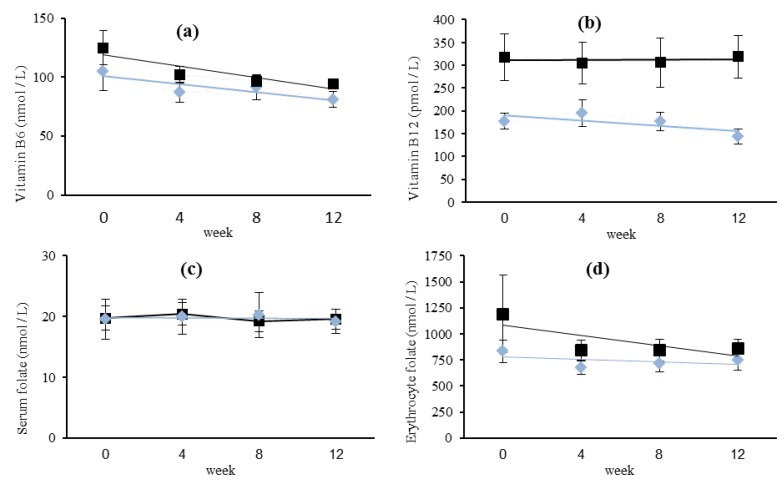
Serum vitamin B_6_ (**a**), vitamin B_12_ (**b**) and folate (**c**), and erythrocyte folate (**d**) concentrations (mean ± SE) at four time-points for OCP ^a^ users 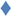 (*n* = 9) and non-OCP users ■ (*n* = 13). Missing data for one OCP user at four weeks.

^a^ Combined OCP containing ethyl estradiol (30−35 mcg) and drospirenone (3 mg); * *P* = 0.042.

Regression analysis showed that vitamin B_12_ concentrations over four time-points was determined by BMI (*P* = 0.031) and when tested by repeated-measures ANOVA, serum vitamin B_12_ values showed a significant difference between OCP users and non-users (*P* = 0.042) (Figure 2). Serum vitamin B_6_, showed a linear decline over time (*P* = 0.017) irrespective of OCP use. Serum vitamin B_6_, folate and erythrocyte folate concentrations did not show any relationships with their respective dietary intake, BMI, age, alcohol intake, MET or haemoglobin concentrations (data not shown).

### 3.2. Dietary Analyses

**Figure 3:**
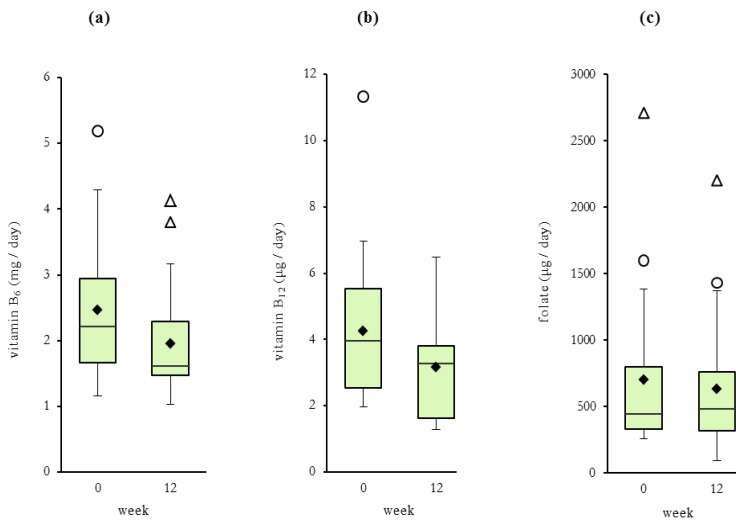
Dietary intakes of vitamins B_6_ mg/day (**a**), B_12_ μg/day (**b**) and folate μg/day (**c**) at weeks 0 and 12. Data shown as boxplots and includes mean (♦) and outliers (○, ∆).

In Table 2 individual mean vitamin intakes are presented for both FFQ (weeks 0, 12). Paired *t*-tests showed no significant differences between week 0 and week 12 for either energy or vitamin intakes. However the absolute intake of vitamin B_6_ was significantly higher at week 0 compared to week 12 (+0.52 ± 0.61 mg/day, *P* = 0.001) (Figure 3). The mean intakes for all vitamins were above the recommended dietary intakes (RDI) however intakes less than the RDI were reported for vitamin B_6_ (13.6%), vitamin B_12_ (36.3%) and folate (36.3%); and less than the estimated average requirements (EAR) for vitamin B_12_ (31.8%) and folate (22.7%). There were no significant differences found between OCP and non-OCP users for mean alcohol intakes (data not shown).

For vitamin B_12_ intakes no significant difference was found between OCP (2.79 ± 1.40 μg) and non-OCP (3.43 ± 1.66 μg) users during the study (source FFQ week 12).

The foods that contributed the intakes of vitamins B_6_, B_12_ and folate are ranked in Table 3. These included energy drinks as a major provider of vitamin B_12_, and reduced fat milk for vitamin B_6_ and folate.

**Table 2:** Participant age, BMI and intakes energy (MJ/day) and vitamins B_6_ (mg/MJ), vitamin B_12_ (μg/MJ) and folate equivalents (μg/MJ) at week 0 and week 12.

			week 0				week 12			
				Daily Vitamin Intake/MJ			Daily Vitamin Intake/MJ			
ID *n* = 22	Age year	BMI kg/m^2^	Energy MJ/day	Vit B_6_ mg	Vit B_12_ μg	Folate Eq μg	Energy MJ/day	Vit B_6_ mg	Vit B_12_ μg	Folate Eq μg
i	18.5	24.3	22.6	0.19	0.31	31.38	16.3	0.14	0.22	37.14
ii	24.8	23.0	19.4	0.19	0.31	69.90	17.3	0.18	0.21	79.43
iii	26.3	19.5	15.8	0.10	0.14 ^a^	34.57	11.7	0.10 ^b^	0.14	38.47
iv ^c^	25.9	25.4	9.5	0.24	0.39	168.56	8.0	0.25	0.54	95.35
v ^c^	27.1	24.7	9.3	0.18	0.27	27.68	8.9	0.17	0.14	85.73
vi ^c^	32.0	20.6	10.7	0.15	0.53	37.59	6.8	0.15 ^b^	0.32	56.56
vii ^c^	20.0	23.3	10.7	0.17	0.22 ^a^	26.75	12.5	0.17	0.21	25.63
viii	23.1	19.0	12.2	0.19	0.48	223.19	7.7	0.21	0.60	287.99
ix	30.2	22.0	7.5	0.24	0.55	36.14	3.2	0.37 ^a^	0.49	28.78
x ^c^	20.0	25.5	8.6	0.18	0.31	37.77	8.1	0.19	0.43	37.89
xi ^c^	21.8	21.7	22.5	0.16	0.25	61.63	24.4	0.16	0.21	58.86
xii ^c^	22.0	22.0	13.7	0.17	0.38	32.74	9.4	0.17	0.37	27.47
xiii	22.2	21.2	9.0	0.23	0.45	94.10	7.3	0.20	0.42	100.13
xiv	24.8	19.7	14.5	0.15	0.27	28.55	13.4	0.15	0.13	28.26
xv	26.8	22.8	11.3	0.10 ^a^	0.19 ^a^	38.62	14.5	0.10	0.26	36.19
xvi ^c^	33.7	21.0	8.7	0.18	0.23 ^b^	43.87	7.0	0.19	0.19	90.79
xvii	25.3	24.5	12.9	0.20	0.31	53.24	11.0	0.18	0.26	38.07
xviii ^c^	34.1	22.6	10.7	0.17	0.21 ^a^	30.56	9.1	0.15	0.15	39.75
xix	24.1	18.7	5.9	0.61	0.44	55.73	6.2	0.25	0.61	30.84
xx	23.5	20.1	16.0	0.17	0.20	49.83	26.2	0.09	0.24	35.25
xxi	24.3	18.7	25.5	0.20	0.44	21.00	9.6	0.43	0.16	67.98
xxii	24.0	20.3	13.7	0.21	0.39	28.67	10.9	0.21	0.60	15.33
										
Mean	25.2	21.9	13.2	0.20	0.33	56.00	11.3	0.19	0.31	61.00
SD	4.2	2.2	5.3	0.10	0.12	49.04	5.6	0.08	0.17	56.69
Median	24.6	21.9	11.7	0.18	0.31	37.68	9.5	0.18	0.25	38.27
Min value	18.5	18.7	5.9	0.10	0.14	21.00	3.2	0.09	0.13	15.33
Max value	34.1	25.5	25.5	0.61	0.55	223.19	26.2	0.43	0.61	287.99

^a^ = intake < RDI; ^b^ = intake < EAR; ^c^ = OCP user.

**Table 3:** Reported contributors of dietary vitamin B_6_, vitamin B_12_ and folate.

Rank ^a^	Vitamin B_6_		Vitamin B_12_		Folate	
	food	% ^b^	food	% ^b^	food	% ^b^
1	beef and veal	29	beef and veal	19	reduced fat milk	16
2	asian greens	11	lamb	17	tomato (raw)	14
3	carrot	11	egg	13	mixed green salad	13
4	reduced fat milk	9	fish	13	strawberries	13
5	banana	9	energy drinks	12	vegetarian lasagne	13

^a^ where Rank 1 is the highest contributor to total intake; ^b^ percentage contribution to daily vitamin intake.

## 4. Discussion

Analyses were undertaken to gain a better understanding of both the within- and between-subject biological variation of vitamins B_6_, B_12_ and folate in women of reproductive age. The strengths of the current study over earlier studies are: firstly, the collection of longitudinal data from a homogeneous group of free-living women; and secondly, the use of standardised protocols that aimed to minimise pre-analytical and analytical variability. When compared to previous studies the current findings show consistently higher within-subject variability data for vitamins B_6_ and B_12_, and modestly lower variability for serum folate [18,19,20].

Longitudinal data enable the evaluation of the extent of the individual variances. In the present study there were multiple excursions from the reference intervals for all analytes with the exception of erythrocyte folate concentrations, which remained within the reference interval. However for serum vitamins B_6_, B_12_ and folate, participants’ values were outside the reference intervals for 1−3 of the four blood collections. Harris [21] argued for a cumulative reporting system for analytical data to accommodate within-subject variations. The present study suggests that more than three samples are required to account for within-subject variability for serum vitamins B_6_, B_12_ and folate in women of reproductive age. Additionally, the indices of individuality (CV_I_/CV_G_) reported for serum vitamin B_12_ and serum- and erythrocyte-folate were <0.6 suggesting that the reference intervals are insensitive when applied to individual variations [19].

The mean vitamin concentrations were not significantly different across the four time points except for vitamin B_6_ where the initial mean value (week 0) was significantly higher than the sample obtained at week 12. This was supported also by repeated-measures analysis of vitamin B_6_. It is possible that routine eating behaviours of the participants were disrupted due to the commencement of the university semester and this may explain elevated serum vitamin B_6_ concentrations at week 0. A change in activity level was considered possibly sufficient to alter vitamin B_6_ absorption, turnover, metabolism or loss; however, statistical analyses did not identify noteworthy changes in MET [22]. In regression analysis we were unable to identify dietary intake or MET as determinants of variability of vitamin B_6_.

Oral contraceptives that contain a combination of oestrogen and progestin, that were taken by the participants have metabolic effects unrelated to contraceptive functions, such as effects on carbohydrate and lipid metabolism [23]. Previous studies reported lower concentrations of vitamins B_6_, B_12_ or folate in users compared to non-users of OCP [24,25,26]. Green *et al.* [27] could not support earlier findings for folate and reported no lowering in adolescents; however vitamin B_12_ levels of OCP users were 33% lower than non-users. In the current study a relationship was identified between OCP use and serum vitamin B_12_ with significantly lower vitamin B_12_ concentrations in OCP users at all four time points. Wilson *et al.* [28] proposes that the lower vitamin B_12_ is a false indicator of deficiency citing Reidel *et al*. [29], who assessed modern biomarkers of vitamin B_12_ status such as methylmalonic acid, holo-transcobalamin and homocysteine concentrations. These data suggested a redistribution of vitamin B_12_ in OCP users rather than a depletion of vitamin B_12_. Research by Shojania and Wylie supports lower total transcobalamin I and higher transcobalamin III levels in OCP users [30]. Overall the impact of OCP on vitamin B_12_ metabolism remains uncertain [28,31]. Data from the present study, using the reference interval for vitamin B_12_ (150–750 pmol/L) show that 50% of OCP users had concentrations below the reference range but were asymptomatic of deficiency; and secondly, the dietary data show that those who consumed equivalent to the lower quartile vitamin B_12_ intakes were not OCP users. Serum vitamin B_12_ data prior to commencement of OCP use were not available preventing further analysis to ascertain whether lower serum vitamin B_12_ is a consequence of OCP use.

Georgiou *et al*. [32] reported that college students have higher quality diets compared with their employed non-student peers and graduates. In the current study 50% of the participants were consuming nutrients at levels <RDI for two of the three vitamins, and this may be reflective of the wider female student population. In examining the sources of vitamins B_6_, B_12_ and folate in the diet, we identified participants who are consuming foods with modest vitamin contents, in quantities that raise the importance of the food as a key vitamin provider. The established listings of foods that provide quantities of these vitamins are reported in credible sources [3,11] however the dietary data from the present study show that there is limited overlap between these listings and the foods consumed by women in the present study. The substantial intake of reduced-fat gourmet coffee drinks elevates milk to the highest source of folate for the participants and the consumption of caffeinated energy drinks matched fish and egg as important sources of vitamin B_12_. Public health concerns have been raised regarding the widespread and excessive consumption of caffeinated energy drinks by young adults [33]. However results of the current study, taken together with reports of meat avoidance [34] and dieting practices [35] by young women suggest that reducing the consumption of caffeinated energy drinks may further lower vitamin B_12_ intakes and increase the percent of women at risk of vitamin B_12_ deficiency. The sources and dietary intakes of vitamin B_12_ in women warrant further investigation.

## 5. Conclusions

This study provides longitudinal data regarding the within-subject vitamin variability for women of reproductive age who were tertiary students. Biochemical data from the current study reinforce earlier findings that clinical decision-making requires serial collections and adherence to standardised protocols. For serum vitamins B_6_, B_12_ and folate, at least three collections were required for a meaningful estimation of vitamin status to be reached. Relationships were not identified between the serum and erythrocyte vitamin concentrations and their dietary intakes; BMI, MET and alcohol intakes. However OCP users presented with consistently lower serum vitamin B_12_ concentrations independent of their dietary intake. The potential for changes in absorption, turnover or storage of vitamin B_12_ needs clarity to establish the true nutritional vitamin B_12_ status of OCP users.
